# Evolutionary Dynamics of Overlapped Genes in *Salmonella*

**DOI:** 10.1371/journal.pone.0081016

**Published:** 2013-11-29

**Authors:** Yingqin Luo, Fabia Battistuzzi, Kui Lin

**Affiliations:** 1 Center for Evolutionary Medicine and Informatics, The Biodesign Institute, Arizona State University, Tempe, Arizona, United States of America; 2 Center for Infectious Diseases and Vaccinology, The Biodesign Institute, Arizona State University, Tempe, Arizona, United States of America; 3 Department of Biological Sciences, Oakland University, Rochester, Michigan, United States of America; 4 College of Life Sciences, Beijing Normal University, Beijing, China; University of North Carolina at Charlotte, United States of America

## Abstract

Presence of overlapping genes (OGs) is a common phenomenon in bacterial genomes. Most frequently, overlapping genes share coding regions with as few as one nucleotide to as many as thousands of nucleotides. Overlapping genes are often co-regulated, transcriptionally and translationally. Overlapping genes are also subject to the whims of evolution, as the gene overlap is known to be disrupted in some species/strains and participating genes are sometimes lost in independent lineages. Therefore, a better understanding of evolutionary patterns and rates of the disruption of overlapping genes is an important component of genome structure and evolution of gene function. In this study, we investigate the fate of ancestrally overlapping genes in complete genomes from 15 contemporary strains of *Salmonella* species. We find that the fates of overlapping genes inside and outside operons are distinctly different. A larger fraction of overlapping genes inside operons conserves their overlap as compared to gene pairs outside of the operons (average 0.89 vs. 0.83 per genome). However, when overlapping genes in the operons separate, one partner is lost more frequently than in those separated genes outside of operons (average 0.02 vs. 0.01 per genome). We also investigate the fate of a pan set of overlapping genes at the present and ancestral nodes over a phylogenetic tree based on genome sequence data, respectively. We propose that co-regulation plays important roles on the fates of genes. Furthermore, a vast majority of disruptions occurred prior to the common ancestor of all 15 *Salmonella* strains, which enables us to obtain an estimate of disruptions between *Salmonella* and *E. coli*.

## Introduction

Genome sequencing technologies are now rapidly producing a large number of microbial genomes [1], [2]. Within the last 15 years, the availability of molecular data has increased from a single complete genome (*Haemophilus influenza*; [3], to thousands of microbial genomes that will soon exceed 3,000 [4]. These genome sequence data are providing opportunities to better understand sequence and genome structure evolution [5], [6], [7], [8], [9]. In particular, comparative genome analyses of closely related species are giving us a unique opportunity to investigate the microevolution of genome structures [10], [11]. One such question pertains to the evolution of overlapping genes (OGs) in bacteria, as many genes have been found to share genomic stretches of their coding regions.

Overlapping genes are a common feature of microbial genomes with more than 30% of the genes showing genomic overlaps [12], [13]. Evolutionary trends of gain and loss of these genes are not yet well understood and their functional importance remains enigmatic [12], [14], [15], [16], [17], [18], [19]. Some consider gene region overlaps to be useful to organisms because shorter genomes are needed to contain all the genes [20], [21], [22]. Others suggest that gene overlaps mitigate detrimental effects of mutation, as selection pressures from multiple genes will remove mutations in the overlapped regions [22], [23].

Irrespective of the evolutionary or selective importance, sequence overlap directly impacts the functional characteristics of the genes involved both at transcriptional and translational levels. When overlapped, gene expression and translation is coupled, which is sometimes considered to be important for coordinated regulation and/or subsequent protein-protein interaction [16], [24], [25], [26], [27].

In bacteria, a large fraction of overlapping genes is found within operons, which are important and well-defined functional units in microbes. Genes in operons are usually co-transcribed and often encode functionally linked proteins [28], [29]. Functional coupling is an efficient way for regulation, especially for complex regulation, because coupling of functionally related genes with one complex promoter would arise more rapidly than two independent complex promoters. In other words, functional coupling results in more economical and efficient mechanisms for functioning [30], [31].

As a genomic tool, overlapping genes are able to display principles of genome evolution, such as indicating phylogenetic relationship among prokaryotes [32], [33]. Interestingly, not all overlapping genes in one species are found to be overlapping in another species. This is true not only for closely related species, but also for strains of a species [13], [34]. This means that new gene couplings can be established and existing ones disrupted by genome-scale mutations [12], [35]. Knowledge of origins of new overlaps and the decay of existing overlaps is useful to gain insights into the evolution of operons and patterns of regulation and gene expression. Therefore, we conducted an analysis of genes that have been historically coupled in the complete genomes of 15 strains of *Salmonella* and constructed a system for querying gain and loss of any pair of overlapping genes in a lineage on a predetermined phylogenetic tree.

## Materials and Methods

### Genome data

We downloaded 15 *Salmonella*, 1 *Shigella*, and 2 *E. coli* genomes from the NCBI database (ftp://ftp.ncbi.nih.gov/genomes/Bacteria; September 2008; Table 1). Overlapping genes were identified from genome structure annotations using in-house PERL scripts. There were a total of 14,295 gene pairs in 18 genomes, excluding 12 genes that had ambiguous start positions. In order to minimize the effect of artificial error during the identification of overlapping genes, we also removed the genes that were annotated with different start or stop sites from their homologous genes in either of two well-annotated model organisms *Salmonella Typhimurium* LT2 (STym) or *Salmonella Typhi* CT18 (STyiCT18) [36], [37]. Of these, 86% genes were translated in the same direction [→→] from the RNA transcript, and the rest showed opposite directions (10.7% facing each other [→←] and 3. 3% facing away from other [←→]).

**Table 1:** Completely sequenced genomes analyzed in this study.

Species	Brief name of species	Accession No.	Genome length (bp)	No. of cds	No. of overlapping genes within *E. coli* operons (pair)	No. of overlapping genes outside of *E. coli* operons (pair)
*S.* typhimurium LT2	*S*Tym*	NC_003197	4857432	4423	661	114
*S.* Typhi Ty2	*S*TyiTy2*	NC_004631	4791961	4318	649	96
*S.* Typhi str. CT18	*S*TyiCT18*	NC_003198	4809037	4395	682	97
*S.* Schwarzengrund str. CVM19633	*S*Sch	NC_011094	4709075	4500	625	158
*S.* Paratyphi C strain RKS4594	*S*PtyC	NC_012125	4833080	4578	566	125
*S.* Paratyphi B str. SPB7	*S*PtyB	NC_010102	4858887	5592	802	373
*S.* Paratyphi A str. ATCC 9150	*S*PtyAATCC*	NC_006511	4585229	4093	578	106
*S.* Paratyphi A str. AKU_12601	*S*PtyAAKU	NC_011147	4581797	4078	555	102
*S.* Newport str. SL254	*S*New	NC_011080	4827641	4612	676	154
*S.* Heidelberg str. SL476	*S*Hei	NC_011083	4888768	4650	659	150
*S.* Gallinarum str. 287/91	*S*Gal	NC_011274	4658697	3965	538	94
*S.* Enteritidis str. P125109	*S*Ent	NC_011294	4685848	4206	594	104
*S.* Dublin str. CT_02021853	*S*Dub	NC_011205	4842908	4514	662	154
*S.* Choleraesuis str. SC-B67	*S*Cho*	NC_006905	4755700	4413	614	142
*S.* Agona str. SL483	*S*Ago	NC_011149	4798660	4562	613	170
*E. coli* str. K-12 substr MG1655	*ECol*K12*	NC_000913	4639675	4295	556	153
*E. coli* O157_H7 str. Sakai	*ECol*O157*	NC_002695	5498450	5230	877	163
*S. flexneri* 2a str. 2457T	*S*Fle*	NC_004741	4599354	4061	623	110

* Genomes with pre-identified orthlogous clusters in ATGC [10].

For each gene in *Salmonella*, we identified a putative ortholog in the *E. coli* genome by using reciprocal BLAST (threshold *E* < 10^−4^ and > 40% similarity). *E. coli* K-12 genome was used as a reference genome because its genome annotation is the most accurate and almost all of its operons have been examined experimentally [38], [39], [40].

As a first approximation, we assumed that pairs of overlapping genes in *E. coli* represent the ancestral state for their orthologs in the *Salmonella* genomes. Based on this assumption, all *Salmonella* genes with orthologs in *E. coli* overlapping genes were categorized into four different configurations. Genes were called coupled (*C*) if the *E. coli* overlapping genes were also overlapping in the given *Salmonella* genome. They were called separated (*S*) if the *E. coli* overlapping genes were no longer overlapping in the *Salmonella* genomes. If only one of the two *E. coli* genes was found in *Salmonella*, then we call it widowed (*W*). If neither *E. coli* gene had a homolog in the given *Salmonella* species, then we refer to the pair as being dead (*D*).

For analyses involving *E. coli* operons, we retrieved *E. coli* K-12 from RegulonDB (version 6.4), which stores comprehensive and highly confident information of operons in *E. coli* [40]. For overlapping genes in other genomes, we used the operons determined in the Database of prOkaryotic OpeRons (DOOR; http://csbl1.bmb.uga.edu/OperonDB) [41].

### Phylogenetic inference

We used the outgroup genome *Yersinia pestis* CO92 (YPes) to root the tree of 15 *Salmonella* strains, *E. coli* K-12 (EColK12), *E. coli* O157:H7 (EColO157), and *Shigella flexneri* (SFle), which was derived from a set of reliable orthologous clusters across genomes. To construct these clusters we first obtained a set of orthologs from 8 genomes (see Table 1) from ATGC database, which identifies orthologs between closely-related microbial genomes [10]. Then, we conducted reciprocal BLAST searches in our genomes missing from the ATGC database. We only included genes that share a high degree of amino acid similarity (*E* < 10^−10^ and > 80% similarity) to take a conservative approach. All “hypothetical”, “unknown”, and “putative” genes were excluded in order to generate clusters of genes with known functions only.

Because phylogenetic analysis can be misled by genes with extremely different GC contents within and among species, we excluded all genes whose GC content showed outlier tendencies as compared to the other orthologs using the Grubb’s test [42]. We also constructed gene-by-gene multiple sequence alignment using MUSCLE in MEGA5 [43], and then computed synonymous divergence using yn00 model in PAML4 [44]. All genes containing gene pairs with synonymous divergence > 1.5 substitutions per site were excluded, because high sequence divergence among strains or species can mislead phylogenetic inference. Short proteins (<150 amino acids) were also removed.

Even after all these exclusions, the final dataset contained 474 genes and 214,491 codons. These genes were concatenated head-to-tail and the fourfold-degenerate sites across all genomes were extracted in MEGA5 (66,202 sites) for phylogenetic analysis; we focus on fourfold-degenerate sites because *Salmonella* genomes are extremely similar to each other at the protein sequence level. We used Neighbor-Joining, Maximum Likelihood, and Bayesian methods for phylogenetic analysis [43]; [45], [46] with a GTR+G+I model for nucleotide substitution from the report of modelTest [47].

### Ancestral states reconstruction

We determined pan-Overlaps, which are pairs of genes overlapping in at least one of the 18 genomes (15 *Salmonella*, 2 *E. coli* and 1 *Shigella*). Using the phylogeny, we constructed a system for querying ancestral states of pan-Overlaps across genomes. Sequence alignments were constructed by using the four possible configurations (*C*, *S*, *W*, and *D*) of overlapping genes in pan-Overlaps as the state symbols. We then inferred ancestral states using parsimony with a user defined matrix (mymatrix) in PAUP 4.0 [48]. The mymatrix was defined as
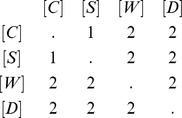



The transforming possibility between *C* and *S* is higher than that among *C* and *W* and *D* because it is rare that widowed and dead overlapping genes become overlapped again as compared with the separated genes to be overlapped during evolution.

### Formation and degradation rates of overlapping genes

To estimate the rate of formation and degradation of overlapping genes, it is necessary to know the number of overlapping genes in *Salmonella* that were newly generated or degraded after diverging from *E. coli*. To obtain the number of newly generated overlapping genes (*n_F_*) in *Salmonella*, we first determined the number of overlapping genes (*n_S_*) contained in each *Salmonella* genome and then calculated the number of pairs of genes (*n_E_*) that are not only overlapping in *E. coli* but also overlapping in *Salmonella*. The number of newly generated *Salmonella* overlapping genes was thus determined as 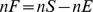.

For the number of newly degraded *E. coli* overlapping genes in *Salmonella*, we first calculated the number of pairs of genes that are overlapping in *E. coli* but have been separated (*n_ES_*) or widowed (*n_EW_*) in *Salmonella*. Since the information on dead overlapping genes have been totally lost in *Salmonella*, we used the number of *E. coli* overlapping genes (*n_ED_*) that do not have any homologous genes in *Salmonella* as a proxy for the number of dead overlapping genes in *Salmonella*. The number of degenerated *E. coli* overlapping genes in *Salmonella* was thus defined as 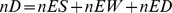.

The rates (*r_iF_* and *r_iD_*) of formation and degradation of overlapping genes in each *Salmonella* genome *i* were then calculated with the formulas 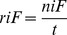 and 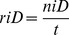 (*i*  =  1 … 15). The divergence time *t* between *E. coli* and *Salmonella* was estimated to be 100 MY [49]. The rates of formation and degradation of overlapping genes (*r_F_* and *r_F_*) were then estimated by the average *r_iF_* and *r_iD_* of the 15 studied *Salmonella* genomes.

### Statistical Tests

Statistical analysis was performed in Graphpad Prism 5.0. A contingency test measured the numbers of *E. coli* coupled overlapping genes inside operons and outside operons that are present or absent as coupled overlapping genes inside operons and outside operons in *Salmonella*.

## Results

### *E. coli* overlapping genes in Salmonella

There are 709 pairs of overlapping genes identified in *E. coli* K-12. At first, we calculated the number of pairs of homologous *E. coli* genes in each of the 15 *Salmonella* genomes. Around one-third (30–35%) pairs have been lost (dead) in each of the *Salmonella* genomes. The remaining *E. coli* overlapping genes (65–70%) have either maintained the overlapping status (coupled) or became non-overlapping (separated or widowed) during the evolution. Of these, most *E. coli* overlapping genes are still coupled in *Salmonella* (from 82.1% to 90.9%), and the remaining *E. coli* overlapping genes have been broken up (8.25–16.8% separated and 1.04–2.12% widowed). The significantly larger fraction of coupled overlapping genes in *Salmonella* indicates that overlapping genes are highly maintained during evolution.

Operons are well defined functional units in microbial genomes. In *E. coli*, a large part of overlapping genes (78.4%) were found within operons, and only 21.6% were outside of operons. Operons may affect the probability of overlapping genes to remain coupled in other genomes during evolution. In order to see whether there is any correlation between overlapping genes and their functional location (inside or outside of operons), we compared the four possible configurations (*C*, *S*, *W*, and *D*) of the *E. coli* overlapping genes inside and outside of operons in the 15 *Salmonella* genomes, respectively. The total number of the homologous counterparts of the *E. coli* overlapping genes in each *Salmonella* genome was defined as the number of all pairs of coupled, separated and widowed overlapping genes in the genome. We were unable to count the number of dead pairs because the information of these *E. coli* overlapping genes has been completely lost in *Salmonella*.

**Figure 1:**
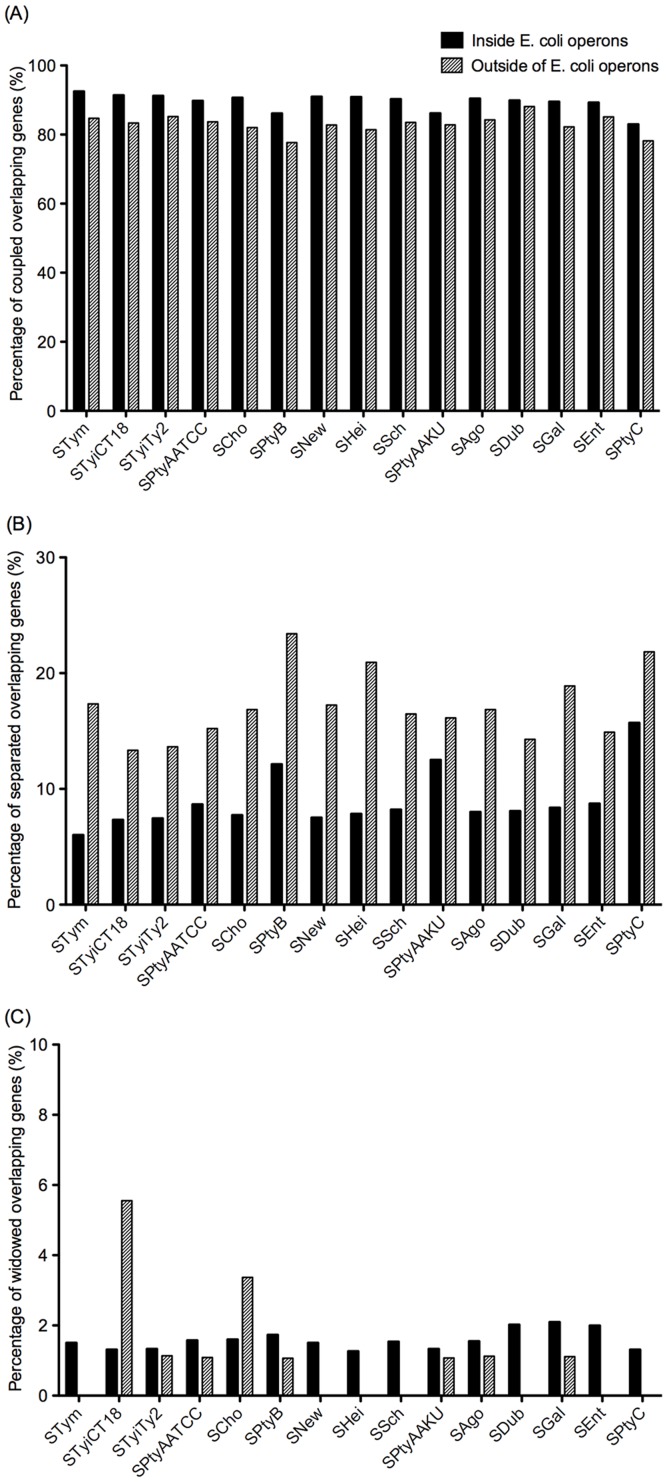
Fate of the *E. coli* overlapping genes in the studied *Salmonella* strains. Percentage of the coupled overlapping genes in *E. coli* that maintain the overlap (A), became separated (B) or became widowed (C) in *Salmonella* genomes.

*Coupled (C).* Fractions of the coupled overlapping genes inside and outside operons were calculated against the total number of orthologs in *E. coli* overlapping genes inside and outside operons. As expected, the fraction of coupled overlapping genes inside operons is consistently larger than that outside of operons (Fig. 1A). The contingency test to each *Salmonella* strain showed that the numbers of coupled overlapping genes inside and outside operons in 12 *Salmonella* genomes have significant difference from the numbers of coupled overlapping genes inside and outside of operons in *E. coli.* Three genomes STyM, SPtyAATCC, and SPtyAAKU didn’t show significant difference. The significant difference was also observed between the numbers of *E. coli* coupled overlapping genes inside and outside operons and the average numbers that are present and absent as coupled overlapping genes inside and outside of operons in *Salmonella* genomes (p-value  =  0.015). These results indicate that overlapping genes inside operons are more likely to remain overlapped because of stronger functional constraints. It was proposed that the formation and decay of overlapping genes were kept at an equivalent frequency in genomes during evolution [12], [15]. We found that overlapping genes outside of operons have lower fraction than that inside operons, suggesting that the formation and decay rates of overlapping genes are not consistent within the genome. The difference between the two groups indicates that stronger purifying selection is acting on the coupled overlapping genes inside operons, as compared with those outside of operons. Some studies have shown that overlapping genes could be used as a phylogenetic marker for the prokaryotic phylogenetic inference [13], [32], [33], [50]. The feature that overlapping genes are more conserved within operons could be used to develop a phylogenetic marker based on overlapping genes.

*Separated (S).* Fractions of the separated overlapping genes against the total number of orthologous counterparts of the *E. coli* overlapping genes were examined in the two groups, inside operons and outside operons, respectively. It was shown that the fraction of separated overlapping genes inside operons is consistently lower than that outside of operons (Fig. 1B). As pointed out previously, operons are important for efficient regulation and optimal expression of genes [51], [52]. On one hand, disruption of the operons could destroy the co-regulation among the complex of proteins and consequentially the transcription efficiency might drastically decrease in those units that lost the promoter [53]. As observed in the coupled overlapping genes, purifying selection is strongly acting on the genes within operons and thus the fraction of separated overlapping gene pairs is much smaller than that outside of operons. On the other hand, although an operon and the individual genes with regulatory structures can be rearranged [52], [53], several lines of evidence suggest that the rearrangement is a conservative process [54]. For example, the rearrangement is constrained by biological pathways [55]. Therefore, due to the strong purifying selection on the genes within operons, decay of overlapping genes is highly restrained within operons in contrast to that outside of operons.

*Widowed (W).* Fractions of the widowed overlapping genes inside and outside of operons were carefully calculated against the total number of orthologous counterparts of *E. coli* overlapping genes in each *Salmonella* genome, respectively. Surprisingly, the fraction of the widowed overlapping genes inside operons is much larger than that outside of operons except for two outliers STyiCT18 and SCho (Fig. 1C). There were no widowed overlapping genes located outside of *E. coli* operons in STym, SNew, SHei, SSch, SDub, SEnt, and SPtyC. Although decay of overlapping genes is highly restrained inside operons as discussed above, it seems that in those cases in which the overlap decays one of the genes is likely to be lost. The second gene inside the operon is located further away from the promoter compared to the first one, and tends to be lost because it is not being transcribed [53]. Alternatively, if either one in the overlapping gene pairs in *E. coli* became a pseudogene [56], the overlapping gene pair was widowed in *Salmonella*. Therefore, the separated *E. coli* overlapping genes were widowed in *Salmonella*. In contrast, genes outside of operons were proposed with no functional coupling. The decay of overlap thus has a smaller impact on the transcription of the second gene outside of the operons than the one inside operons. In other words, both genes are still being transcribed when the overlapping genes are separated and located outside of the operons. The result is also supported by the significantly larger distance between two separated overlapping genes inside operons compared to that of genes outside operons (at the level of 0.05, p-value  =  0.02). However, this pattern does not hold for two of the genomes, STyiCT18 and SCho. One possible reason is that genes might still be co-regulated without being in the same operons. Alternatively, the disruption of overlap does not necessary destroy the structure of operons.

*Dead (D).* Any pair of overlapping genes can be lost in three possible ways: (I) A pair of overlapping genes was entirely deleted from the genome by an evolutionary event. In our case, *E. coli* overlapping genes become dead overlapping genes in *Salmonella* in one evolutionary event. (II) *E. coli* overlapping genes were separated in *Salmonella* by point mutations, and subsequently lost in tandem or became pseudogene(s). (III) Either one of the two individual genes in the overlap was lost, followed by loss of the other one during evolution.

Even though the information of the *E. coli* overlapping genes was totally lost in *Salmonella* when the overlapping genes are dead, it is interesting to know which overlapping genes tend to get lost during evolution. There are 22 and 10 pairs of *E. coli* overlapping genes inside and outside of operons, respectively, that have been lost in all the *Salmonella* genomes. Not surprisingly, most genes that tend to be lost are related to phage genes or insertion elements in *E. coli*. However, there is an obvious difference between the dead genes inside and outside of operons. For example, except for two pairs with unknown functions, genes in the remaining 8 pairs outside of operons were all classified into the functional group ‘Replication, recombination and repair’ based on COG, but the genes inside operons have various functions. Most genes inside operons have clear biological functions (e.g., *glcDEFGBA* is related to Glyoxylate and dicarboxylate metabolism, *alsBACE* is D-allose transporter subunits, and *rpiR* is DNA-binding transcriptional repressor) or are involved in important pathways (e.g., *atoDAEB* is a highly inducible system for acetoacetate and butyrate degradation [57], and *gspCDEFGHIJKLMO* is involved in cryptic general secretory pathway [58] in *E. coli*). Although operons are under strong purifying selection [29], [30], known operons in *E. coli* that have been either particularly or entirely lost in other species indicates that genome rearrangements within operons are not rare in bacterial genomes.

### Pan-Overlaps in Salmonella

**Figure 2:**
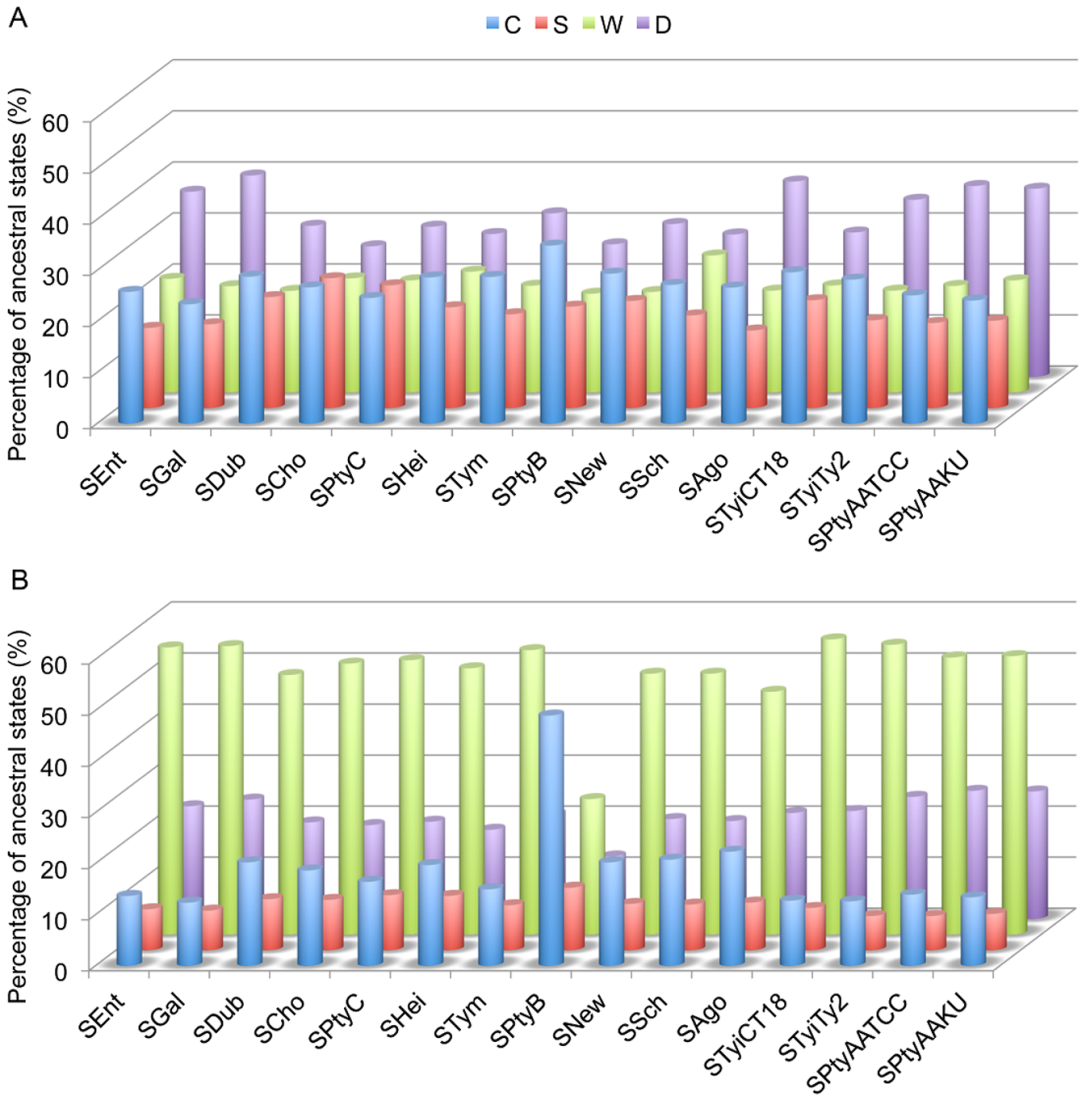
Fate of the genes in pan-Overlaps in *Salmonella* strains. (A) Average of the percentages of the genes inside operons. (B) Average of the percentages of the genes outside of operons.

A set of pan-Overlaps was determined based on all of the overlapping genes identified in the 15 *Salmonella*, two *E. coli* and one *Shigella*. In total, there were 3062 pairs of overlapping genes in the pan-Overlaps containing two groups, 2301 pairs inside operons and 761 pairs outside of operons (See Table S1, the first sheet contains all pan-Overlaps inside operons and the second sheet contains all pan-Overlaps outside operons). As have been defined previously, each pair of overlapping genes in the pan-Overlaps has one of four possible configurations (*C*, *S*, *W*, and *D*). We compared the distribution of the configurations over the 15 *Salmonella* groups (Fig. 2). Percentage of the coupled overlapping genes (*C*) in pan-Overlaps inside operons is much higher than that outside of operons (average 28% and 19%, respectively), suggesting that gene pairs inside operons have greater propensity of overlap as compared with that outside of operons. However, interestingly, although dead overlapping genes (*D*) both inside and outside of operons have high percentages (average 32% and 20%, respectively), *D* inside operons shows the largest percentage among the four configurations, indicating that although overlapping genes are highly maintained within operons, these genes may be subject to the whims of evolution. Paired comparison analysis also shows the significant difference among the four configurations (two tailed t-test at p-value < 0.05). For the gene pairs outside of operons, *W* shows the highest percentage among the configuration (average 52%), which is more than two times higher than that for the genes inside operons (average 21%). The significantly larger percentage of *W* genes compared to *S* genes (two tailed t-test at p-value < 0.0001) implies that genes tend to die (particularly one partner gets lost more frequently than the other one) when the overlaps are broken. The difference between the *W* and the *D* configuration outside of operons (two tailed t-test at p-value < 0.0001) shows that genes outside of operons were widowed more frequently but less frequently lost as compared to those inside operons. We also observed that there is no significant difference between the *C* configuration and the *D* configuration outside of operons, which indicates that coupled overlapping genes outside of operons might not be maintained during the evolution. Separated overlapping genes (*S*) both inside and outside of operons have the smallest percentages among the four configurations (19% and 9%, respectively), indicating that pairs of overlapping genes tend to be lost or widowed rather than kept presenting a new (separated) configuration. In summary, the overlapping genes are highly maintained inside operons as compared with those outside of operons, however, when the overlaps are disrupted, genes inside operons tend to be lost rather than kept as separated genes.

### Phylogeny

**Figure 3:**
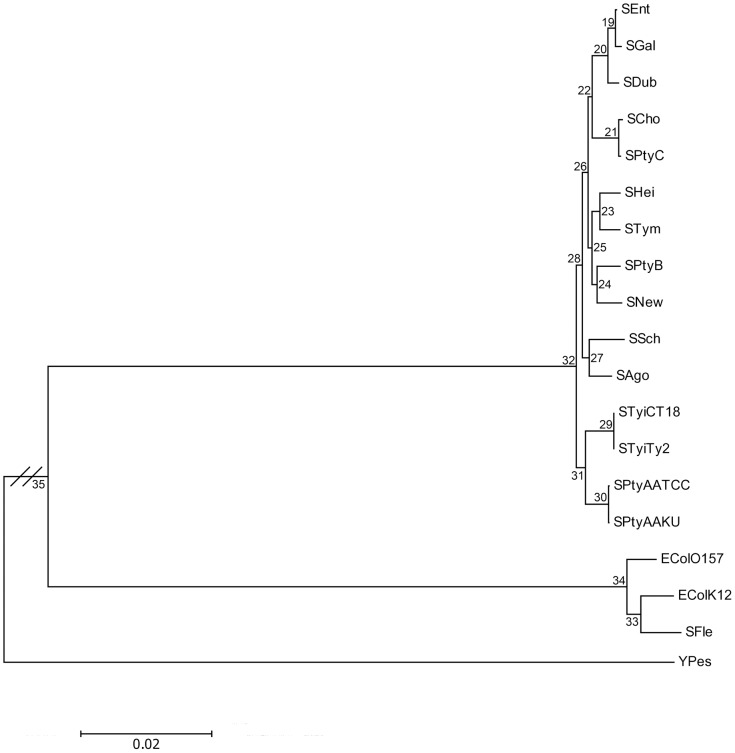
The ML phylogeny inferred from the four-fold degenerate sites of 474 genes. The internal nodes are labeled 19–35, with node 35 being the divergent point between *E. coli* and *Salmonella* and node 32 being the root node of the ingroup *Salmonella*.

The finding of a strong, reliable phylogenetic tree allows the inference of states dynamics of overlapping genes along the history of the species. To determine the ancestral states of the overlapping genes in pan-Overlaps, we firstly determined a phylogenetic tree of the 19 studied genomes (including the outgroup YPes). The phylogenies were obtained using NJ, ML and Bayesian methods based on the four-fold degenerate sites of a concatenated data set of the 474 genes (see 1 for the ML tree, Fig. S2 for the NJ tree, and Fig. S3 for the Bayesian tree). The topologies of the ML, NJ, and Bayesian trees are highly similar except for two taxa (SCho and SPtyC) that show controversial positions with low bootstrap value in the ML tree but high bootstrap values in the NJ and Bayesian trees (Fig. S2, S3). In the ML and Bayesian trees, the group SCho and SPtyC were located close to the tip node (SEnt, SGal, and SDub), a position similar to that found recently in a phylogeny based on 2,898 single-copy genes [59]. Differently, the group SCho and SPtyC was closer to the group (SAgo and SSch) with the taxa STym being more recent in the NJ tree; this position is comparable to that inferred using presence-absence predictions for genes that exhibit inconsistent distributions within *Salmonella* [60]. For this reason, and because the ML and Bayesians trees are in good agreement, despite the poor support of several nodes in the ML phylogeny, we decided to use the ML tree in the following analysis (Fig. S1, Fig. 3).

### Ancestral states of pan-Overlaps

To understand the evolutionary history of overlapping genes, it is necessary to know not only the present character states, but also their ancestral states [61], [62]. Reconstructing ancestral states of overlapping genes from present data can provide a unifying framework for understanding the origins and evolution of overlapping genes. Using the ML tree built based on the four-fold degenerate sites, we reconstructed the possible evolutionary states (*C*, *S*, *W*, and *D*) for each pair of overlapping genes in pan-Overlaps over the studied strains [48] (Fig. 3).

**Figure 4:**
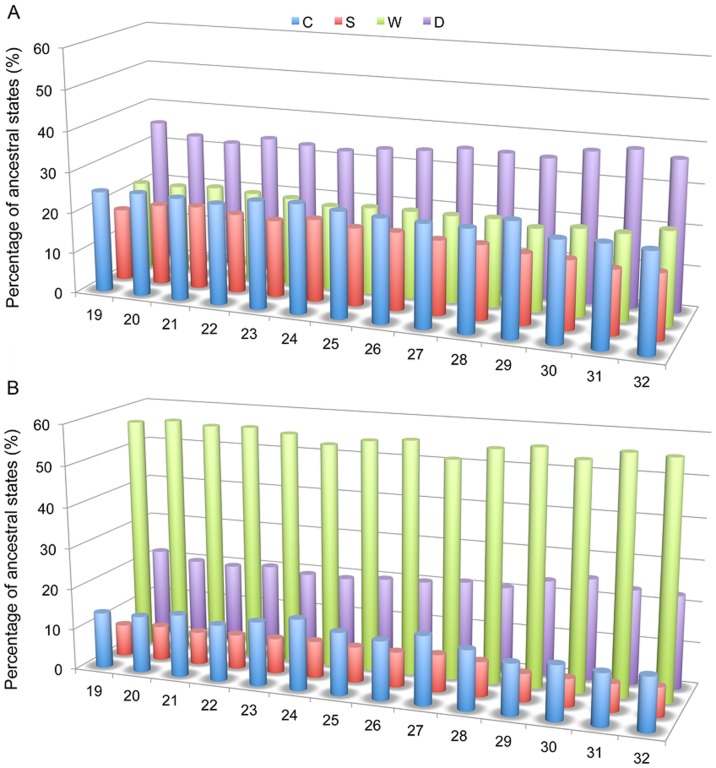
Fate of the gene pairs in pan-Overlaps over the internal nodes of the phylogeny of the studied genomes. (see Fig. 3 for node numbering). (A) Average of the percentages of the gene pairs inside operons over the internal nodes of the phylogeny. (B) Average of the percentages of the gene pairs outside of operons over the internal nodes of the phylogeny.

We compared the distributions of ancestral states of the genes in pan-Overlaps inside operons with that outside of operons. It shows the distribution of ancestral states of pan-Overlaps represents a similar pattern to that of the overlapping genes at the present nodes, but has much more significant variance among the four configurations (Fig. 4). Four configurations *C*, *S*, *W*, and *D* both inside (Fig. 4A) and outside operons (Fig. 4B) show significant variance at two tailed t-test p-value < 0.05). Interestingly, dominance of the four configurations (*C*, *S*, *W*, and *D*) represents the same order at all of the internal in-group nodes (19–32; Fig. 3) both inside (*D* > *C* > *W* > *S*) and outside of operons (*W* > *D* > *C* > *S*). However, Grubbs' test shows that there is no deviation among all of the internal nodes at p-value  =  0.05 level. No significant changes among the internodes means there is no significant propensity of loss of overlapping genes among the internodes. Our study indicates that there is no significant relationship between the formation and decay of overlapping genes and the evolutionary distance among the strains.

### Rates of formation and degradation of overlapping genes

It is obvious that the degeneration and formation of overlapping genes are common events in bacterial genomes. Some researchers have proposed that the degenerate rate might equal the formation rate since there is a very good correlation between the number of ORFs and the number of overlapping genes observed [12]. However, in our study, we found that the overlapping genes inside operons show different evolutionary histories than those outside of operons, suggesting that the evolutionary rate of overlapping genes within the genomes are different. Using the known divergence time between *E. coli* and *Salmonella*, we estimated the rates of formation of overlapping genes as 4.5×10^−9^ and 1.6×10^−9^ per pair per year, inside and outside of operons, respectively. The rates of degeneration of overlapping genes are 4.1×10^−9^ and 2.6×10^−9^ per pair per year, inside and outside of operons, respectively.

Formation of operons might be driven by the horizontal gene transfer [63], [64], or by co-function [30]. It is probable that many overlapping genes were generated during the formation of operons. As we expected, the degeneration rate of overlapping genes inside operons is much lower than that outside of operons, indicating overlapping genes are highly maintained inside operons due to stronger selection compared with the overlapping genes outside of operons.

## Discussion

### Possible influence of dynamic evolution in *E. coli*

In this analysis, we used *E. coli* K-12 as the reference to the *Salmonella*. The discussion about the detected patterns of four configurations (*C*, *S*, *W*, and *D*) of the *E. coli* overlapping genes inside and outside of operons in *Salmonella* genomes is based on the assumption that no dynamic evolution in *E. coli* after the divergence of *Salmonella* from *E. coli*. However, the scenario could be influenced by the dynamic evolution of overlapping genes in *E. coli* because overlapping genes in *E. coli* can also have evolutionary dynamics similar to the detection in *Salmonella* in this study. For example, we found that the relative fractions of dead overlapping genes are higher inside operons than outside of operons. If we considered the dynamic evolution in *E. coli* as in *Salmonella*, which also has a higher rate of formation of overlapping genes inside operons than outside operons, then it would explain that the relative higher fraction of dead overlapping genes inside operons than outside operons in Salmonella. This conclusion would be different from the original explanation that higher fraction of dead overlapping genes inside operons than outside operons is due to the strong purifying selection in operons. Newly formed operons in *E. coli* could also influence the original explanation to the scenario of dead overlapping genes in *Salmonella*. On one hand, because such newly formed operons in *E. coli* will not be found in *Salmonella*, they will be considered as dead in *Salmonella*. On the other hand, it is possible that genes appearing as newly formed ones in *Salmonella* could be originally present in common ancestor of *E. coli* and *Salmonella*, but were afterwards subject to decay in *E. coli*. In this study, we simplified the complex process of evolution. The dynamic evolution in *Salmonella* is addressed based on a basic model that *E. coli* overlapping genes is a static frame of reference, however, the scenarios we discussed here could be influenced due to the evolution of overlapping genes in *E. coli* in parallel to *Salmonella*.

### Function of overlaps

**Figure 5:**
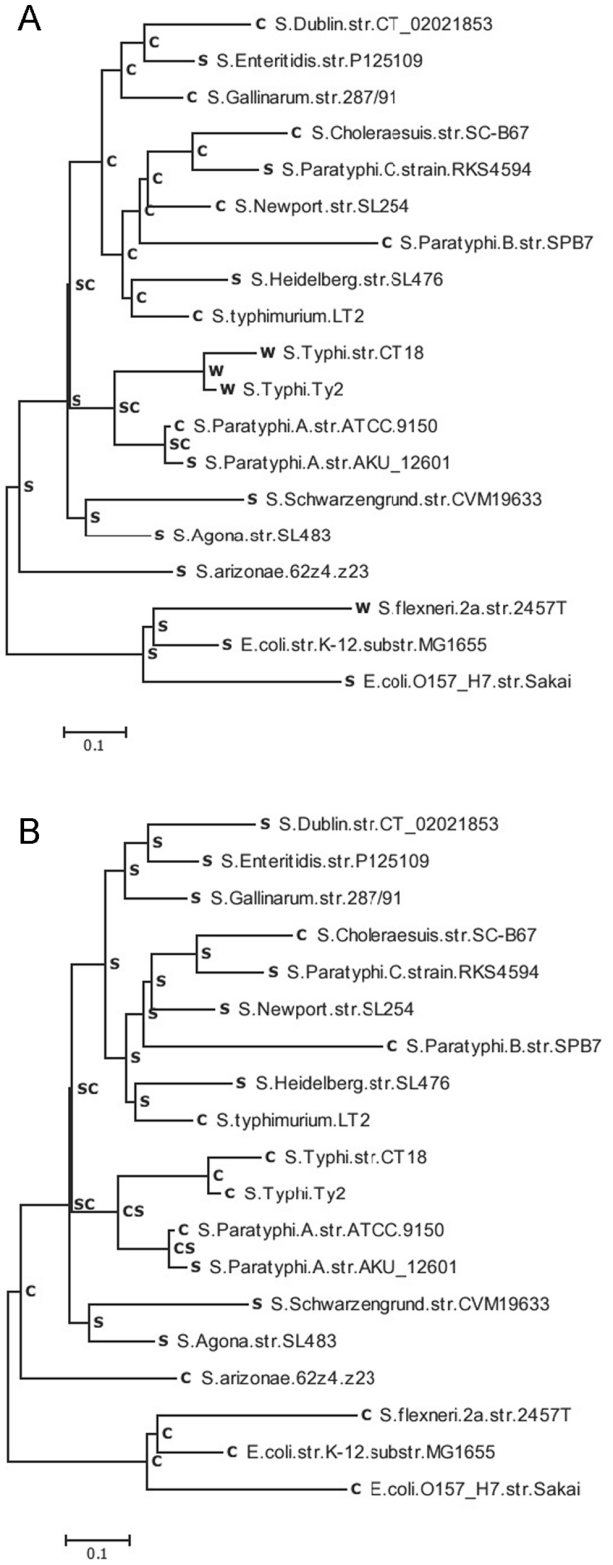
Phylogenetic map of the overlapping gene pairs. (A) Phylogenetic map of the overlapping gene pair (*bcsC*, *bcsZ*). (B) Phylogenetic map of the overlapping gene pair (*ompR*, *envZ*).

It is useful to understand the configuration and evolutionary status of interesting pairs of overlapping genes across a lineage of species because the overlap status of pairs of genes can be used as a phylogenetic trait to infer functions [16], [19]. For example, the couple *bcsC-bcsZ* is linked to cellulose production in those strains and species in which the overlap is maintained. *bcsC* and *bcsZ* are overlapping in the genome STym with the downstream region of *bcsC* overlapping by 19 bp with the upstream region of *bcsZ*, while they are separated in *E. coli* and *S.* Typhi. The two genes are located in the operon bcsABZC, which is a characteristic cellulose biosynthesis operon [65], [66]. As it is known, cellulose production confers bacterial cell-cell interactions, adhesion to abiotic surfaces for biofilm formation and chlorine resistance to the organism [67]. Regulation of cellulose biosynthesis varies widely among species and even within a species. Studies have shed light on cellulose biosynthesis in several bacterial genomes, such as STym and SEnt [66]. *E. coli* and *S.* Typhi do not produce cellulose suggesting that this could be caused by the separation of the *bcsC* and *bcsZ* genes leading to a preterminated out-of-frame product. Therefore, based on the evolutionary status of *bcsC* and *bcsZ* in the phylogentic tree (Fig. 5A), we would predict that strains STym, SDub, SGal, SCho, SNew, SPtyB, and SPtyA produce cellulose and SPtyC, SHei, SPtyAAKU, SSch, SAgo, STyiTy2, and STyiCT18 have no cellulose because the *bcsC-bcsZ* have been separated or widowed. Cellulose expression has variable patterns in *Salmonella* and *E. coli*, but the linkage with the overlap status would be very important information because it provides a bioinformatic approach to test the presence or absence of a functioning pathway.

Another example is the couple *ompR*-*envZ*, which is linked to virulence of the pathogens. In *E. coli*, the upstream gene *ompR* coding for a positive transcription regulator OmpR has 4 bp overlapping with *envZ* coding for an inner membrane protein EnvZ. It is known that the *ompR-envZ* two-component regulatory system is essential for the response to environment signals and contributes to virulence in a number of enteric bacterial pathogens [68], [69]. Phosphorylation of OmpR by osmosensor EnvZ modulates synthesis and normal functioning of the proteins OmpC and OmpF located on the external side of the membrane. It has been shown that the translation efficiency of *envZ* could decrease ten times or more when the translation of *ompR* is terminated away from the normal stop codon [70]. Although the *ompR-envZ* locus is highly conserved within the *E. coli* and *Salmonella* genomes, the two genes show variable configurations across the *Salmonella* genomes (Fig. 5B). Overlap of the two genes is maintained in the *Salmonella* Typhi genomes (*S*TyiTy2 and *S*TyiCT18), STym, SPtyB and SCho, while they have been separated (*S*) in the remaining *Salmonella* genomes. The separated status suggests that genes coding for outer membrane porin proteins OmpF and OmpC would not be normally expressed due to the low translation efficiency of *envZ* that modulates expression of *ompF* and *ompC*. Translational interrelation of the synthesized products of overlapping genes is of practical importance. The phylogenetic analysis of the evolutionary status of overlapping genes would provide researchers with a new tool to study the roles of *ompR-envZ* in the regulation of genes, especially in the virulence secretion system.

## Supporting Information

Figure S1 **The ML tree inferred from the four-fold degenerate sites of 474 genes with 100 bootstrap replicates.**
(TIF)

Figure S2 **The NJ tree inferred from the four-fold degenerate sites of 474 genes with 100 bootstrap replicates.**
(TIF)

Figure S3 **The Bayesian tree inferred from the four-fold degenerate sites of 474 genes with 100 bootstrap replicates.**
(TIF)

Table S1 **The overall set of pan-Overlaps in** ***Salmonella*** **and** ***E. coli*** **.** The first sheet in the Table S1 contains all pan-Overlaps inside operons and the second sheet in the Table S1 contains all pan-Overlaps outside operons.
(XLSX)
