# Supplementary figures and images for: Evolutionary Dynamics of Overlapped Genes in Salmonella
Source: PLoS One. 2013 Nov 29;8(11):e81016. doi: 10.1371/journal.pone.0081016 (PMC3843671; doi:10.1371/journal.pone.0081016)

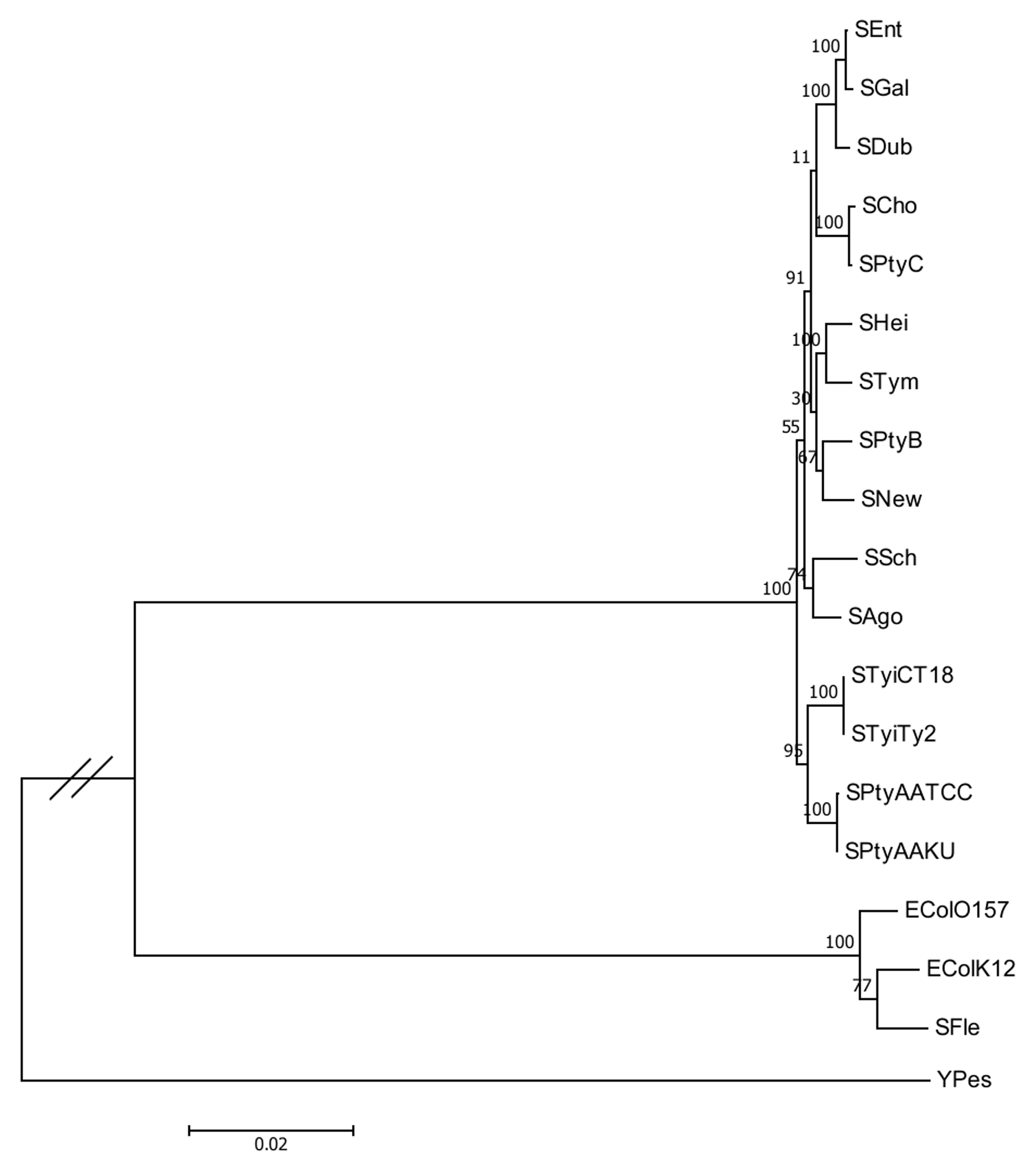

Supplement: Figure S1 — The ML tree inferred from the four-fold degenerate sites of 474 genes with 100 bootstrap replicates. (TIF) [file pone.0081016.s001.tif]

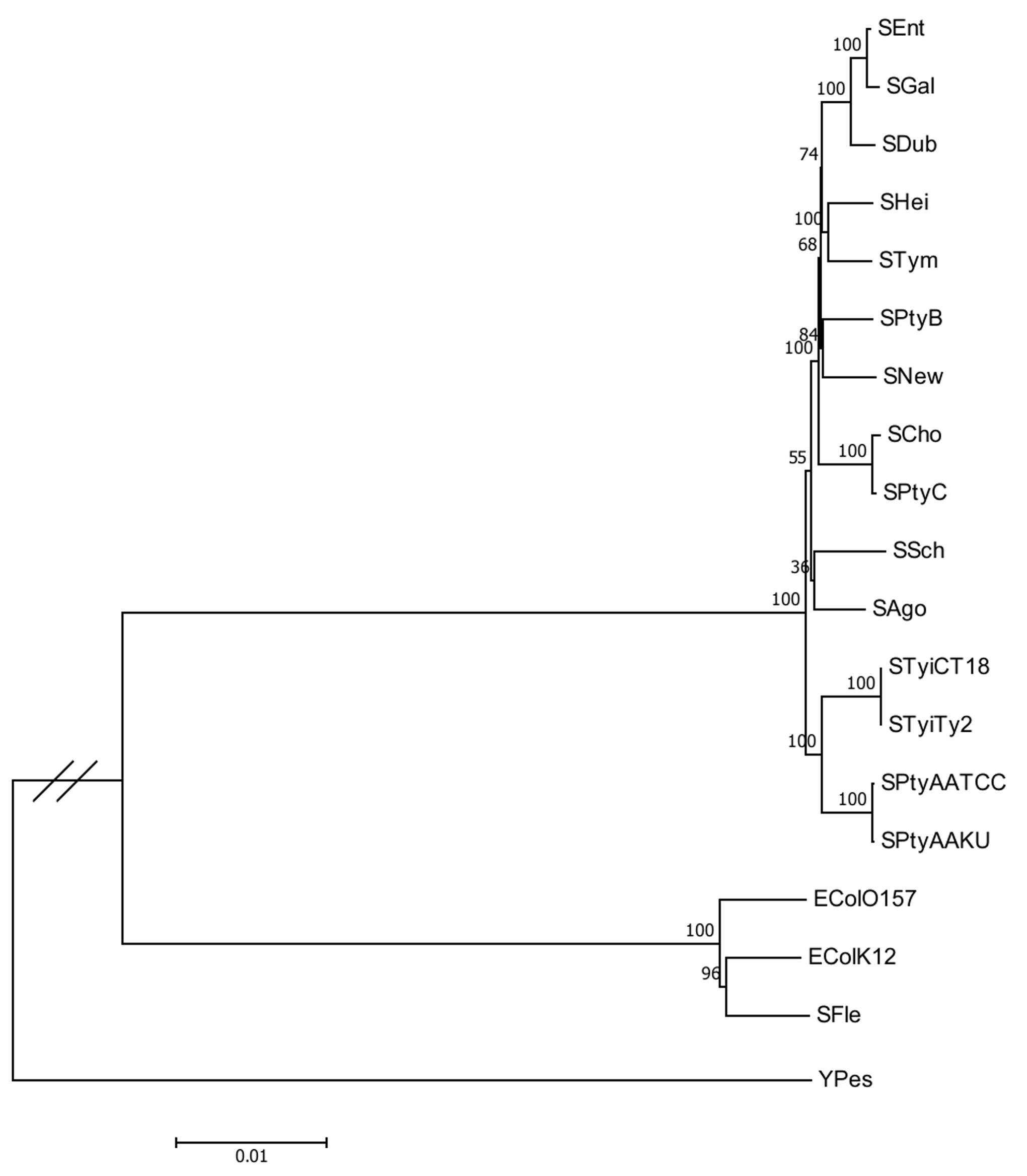

Supplement: Figure S2 — The NJ tree inferred from the four-fold degenerate sites of 474 genes with 100 bootstrap replicates. (TIF) [file pone.0081016.s002.tif]

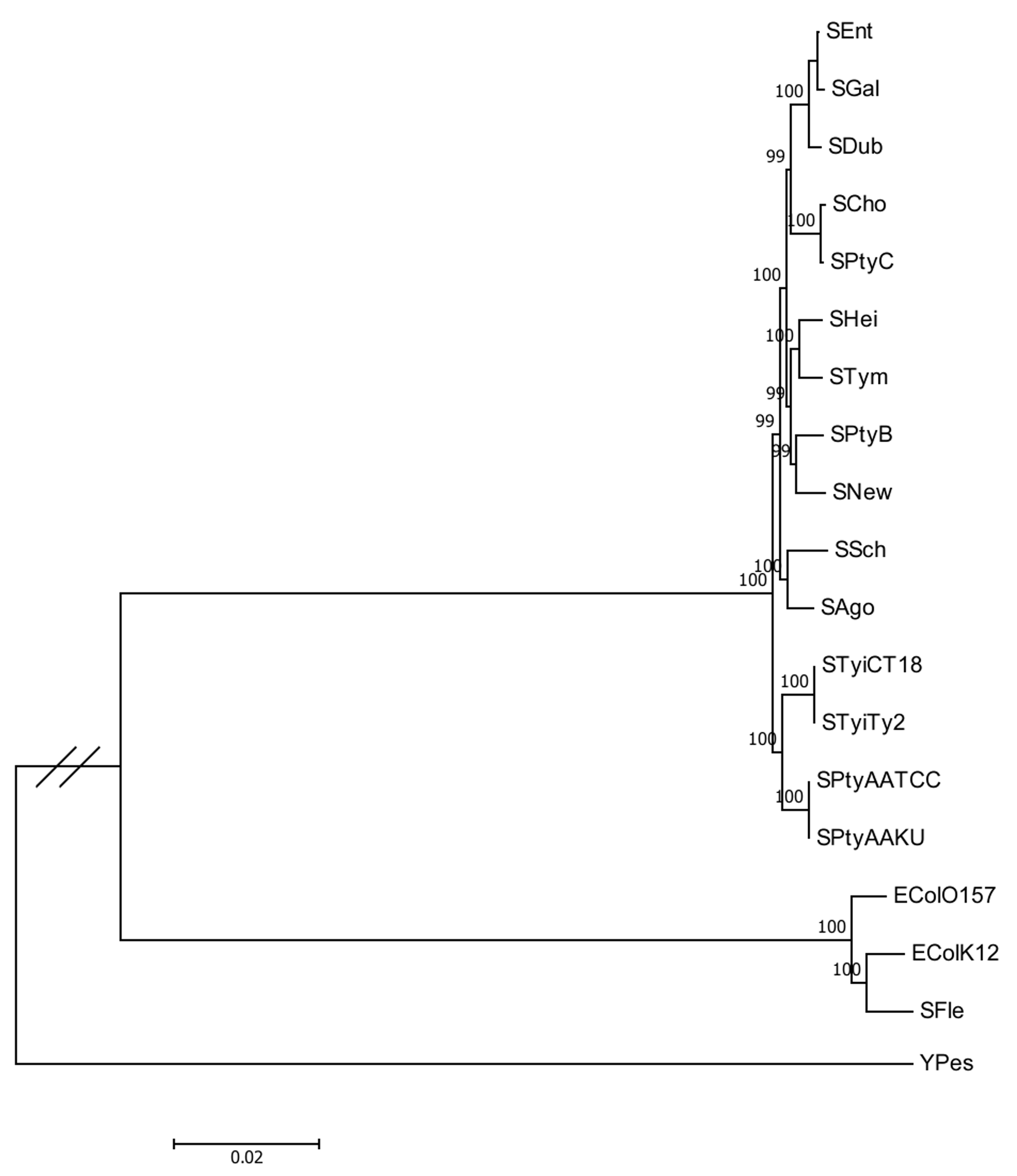

Supplement: Figure S3 — The Bayesian tree inferred from the four-fold degenerate sites of 474 genes with 100 bootstrap replicates. (TIF) [file pone.0081016.s003.tif]
